# NOTCH regulation of the endothelial cell phenotype

**DOI:** 10.1097/MOH.0000000000000425

**Published:** 2018-04-05

**Authors:** Julia J. Mack, M. Luisa Iruela-Arispe

**Affiliations:** a Department of Molecular, Cell and Developmental Biology; b Molecular Biology Institute, University of California, Los Angeles, California, USA

**Keywords:** angiogenesis, atherosclerosis, cardiovascular, vessels

## Abstract

**Purpose of review:**

The formation of a hierarchical vascular network is a complex process that requires precise temporal and spatial integration of several signaling pathways. Amongst those, Notch has emerged as a key regulator of multiple steps that expand from endothelial sprouting to arterial specification and remains relevant in the adult. This review aims to summarize major concepts and rising hypotheses on the role of Notch signaling in the endothelium.

**Recent findings:**

A wealth of new information has helped to clarify how Notch signaling cooperates with other pathways to orchestrate vascular morphogenesis, branching, and function. Endothelial vascular endothelial growth factor, C-X-C chemokine receptor type 4, and nicotinamide adenine dinucleotide phosphate oxidase 2 have been highlighted as key regulators of the pathway. Furthermore, blood flow forces during vascular development induce Notch1 signaling to suppress endothelial cell proliferation, enhance barrier function, and promote arterial specification. Importantly, Notch1 has been recently recognized as an endothelial mechanosensor that is highly responsive to the level of shear stress to enable differential Notch activation in distinct regions of the vessel wall and suppress inflammation.

**Summary:**

Although it is well accepted that the Notch signaling pathway is essential for vascular morphogenesis, its contributions to the homeostasis of adult endothelium were uncovered only recently. Furthermore, its exquisite regulation by flow and impressive interface with multiple signaling pathways indicates that Notch is at the center of a highly interactive web that integrates both physical and chemical signals to ensure vascular stability.

## INTRODUCTION

The process of blood vessel formation requires strict coordination of proliferation, differentiation, and maturation. These are concurrent events in a vascular sprout and require the integration of multiple signaling pathways, including but not limited to: vascular endothelial growth factor (VEGF), bone morphogenetic proteins (BMPs), and Notch [1,2].

Highly conserved across vertebrate species, the Notch signaling pathway includes a group of four transmembrane receptors (Notch1–4) that interact with five transmembrane ligands (Jagged1,2; Delta-like 1, 3, and 4). Activation involves two sequential proteolytic events that require the enzymes a disintegrin and metalloproteinase domain-containing protein 10 and γ-secretase [3]. Cleavage of Notch results in the release of its intracellular domain (ICD) from the membrane and its subsequent translocation to the nucleus where it acts as a transcriptional coactivator through cooperation with recombination signal-binding protein for immunoglobulin kappa J (RBPJ) [4]. As a transcriptional regulator, NOTCH ICD (NICD) controls expression of a cohort of target genes that impact cell physiology [5,6].

The influence of Notch signaling in differentiation and development cannot be overstated as Notch regulates fate decisions in cell types of all four major tissues: epithelium, muscle, connective (specifically hematopoietic cells and bone in this group), and nervous tissue [4]. During development, Notch is known for its ability to generate cell and tissue boundaries by promoting the emergence of distinct differentiation programs from an apparently homogenous group of cells. This function relates to its well known role in lateral inhibition that works through a positive feedback loop. In the vascular system, Notch is known to coordinate tip versus stalk cell fates within a growing sprout. Essentially, expression of Delta-like 4 (Dll4) in the cell at the end of the sprout (tip cell) activates Notch1 in the stalk cell. In this manner, the formation of vascular sprouts requires a highly calibrated temporal and spatial localization of Notch signaling. Recent findings have expanded our understanding of the integration of Notch signaling with other pathways in the formation, remodeling, and homeostasis of the vascular tree. This review focuses on key advances in our understanding of how Notch influences blood vessels.

### Regulation of tip and stalk cell: a dynamic dance

**FIGURE 1:**
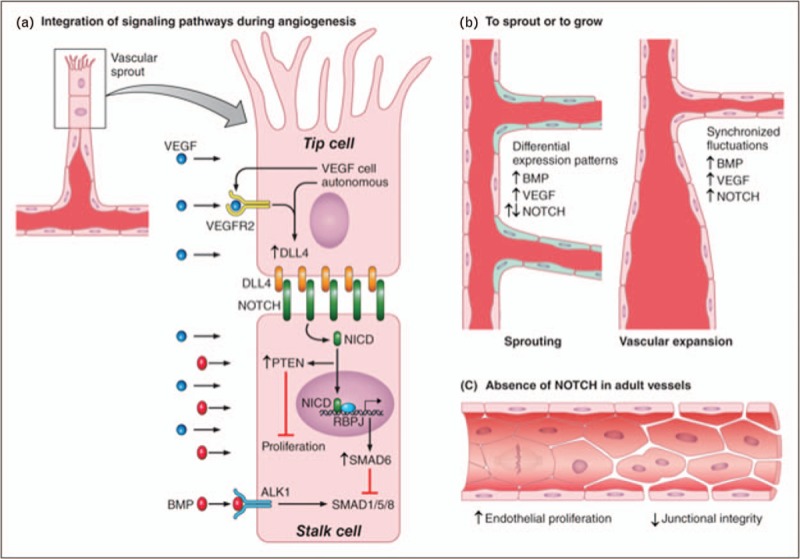
NOTCH signaling in the endothelium. (a) A vascular sprout is characterized by a leading ‘tip’ cell followed by ‘stalk’ cells. Tip cells express high levels of DLL4 that activate NOTCH1 in the stalk cells to promote NOTCH signaling and impose differential gene expression. Increase of DLL4 in tip cells is achieved by both endothelial and non-endothelial derived VEGF to activate VEGFR2. In stalk cells, activation of the receptor results in generation of NICD that translocates to the nucleus where it binds to RBPJ and regulates gene expression. A consequence of NOTCH activation is to increase PTEN levels that suppress proliferation and to upregulate SMAD6 that titrates the signaling mediated by BMPs. (b) Regulation of new sprouting during vascular expansion depends on integration of BMP signaling, NOTCH signaling and VEGF signaling. Differential expression patterns (illustrated by the different colors in the schema) of the NOTCH, VEGF, and BMP pathways are required to enable sprouting of new vessels. In contrast, synchronized fluctuations of the pathways favor vessel enlargement and disfavor branching. (c) In adult vessels, NOTCH is responsible for maintaining endothelial quiescence and junctional integrity. BMPs, bone morphogenetic proteins; DLL4, delta-like 4; NICD, NOTCH ICD; PTEN, phosphatase and tensin homolog; RBPJ, recombination signal-binding protein for immunoglobulin kappa J; SMAD, mothers against decapentaplegic; VEGF, vascular endothelial growth factor; VEGFR2, vascular endothelial growth factor receptor 2.

Regulation of cell fate decisions is a hallmark of Notch signaling, and in the vasculature, the first effect of Notch is to promote stalk/tip cell specification. Pharmacological or genetic inactivation of Notch signaling at the onset of angiogenesis results in a remarkable expansion of tip cells at the expense of stalk cells. The outcome of Notch inactivation is loss of vascular hierarchy as tip cells are unequipped to organize tubes or form stable junctional complexes. The phenotype explains why inhibition of Notch in tumors results in suppression of tumor growth despite a high number of endothelial cells and sprouts [1]. The collective body of knowledge indicates that Notch is required for vascular stabilization and differentiation of the emerging vascular tree and it does so through suppression of endothelial cell proliferation and stabilization of cell–cell junctions [7,8^▪▪^,^9^^▪▪^]. How Notch blocks proliferative signals has been unclear until recently when Notch-mediated inhibition of proliferation was found to require the well known tumor suppressor phosphatase and tensin homolog (PTEN) [10]. Using both gain and loss-of-function approaches, the authors demonstrated that PTEN is crucial for blocking stalk cell proliferation downstream of Notch signaling (Fig. 1a).

Notch signaling is also required for proper formation of vascular branches. Recent work demonstrated that endothelial branching in response to BMP signaling and activation of mothers against decapentaplegic (SMAD)1/5/8 are Notch dependent. Specifically, SMAD6, an inhibitory SMAD that titrates input signals from SMAD1/5/8, is regulated cell intrinsically by Notch levels [11^▪▪^] (Fig. 1a). In this manner, the distribution of vascular sprouts depends on the ‘Notch status’ of a given cell within the length of the sprout. Furthermore, the decision to either form a new sprout or widen the original vessel relies on differential expression patterns of Notch-Dll4, BMP, and VEGF between cells (Fig. 1b). Real-time visualization of Dll4 levels in a new sprout showed uncoordinated fluctuations that can be quickly synchronized by VEGF-A. This, in turn, causes a potential sprout to retract, allowing for the original vessel to widen [12^▪^]. Therefore, it is the integration of Notch signaling, BMP, and VEGF that coordinate expansion in sprouting or vessel diameter (Fig. 1b). Endothelial VEGF-A and subsequent activation of vascular endothelial growth factor receptor 2 (VEGFR2) are essential to induce Dll4 and Notch-dependent vascular growth in the adjacent cell [12^▪^,^13^^▪▪^]. Deletion of either VEGF-A or VEGFR2 in endothelial cells regulates levels of Dll4 in tip cells and, in turn, Notch inhibition provides a feedback loop that reinforces expression of VEGF-A and C-X-C chemokine receptor type 4 (CXCR4), which stimulate endothelial sprouting and proliferation in the expanding vascular plexus [13^▪▪^].

Our understanding of the molecular signals that regulate Notch during vascular sprouting has broadened with the identification of upstream regulators that include nicotinamide adenine dinucleotide phosphate (NADPH) oxidase 2 (Nox2) and the endothelial transcription factor ETS-related gene (Erg) [14^▪^,15^▪^]. Erg was shown to control the balance of Notch ligands Dll4 and Jagged1 (Jag1) as well as the levels of Notch modulators Manic fringe and Lunatic fringe (Lfng), which directly affect vascular development. Recent investigations into the balance of Notch ligands during angiogenesis has uncovered an interaction between Jag1 and the intermediate filament vimentin that may act to titrate specific ligand signaling strengths [16]. Using induced pluripotent stem (iPS) cells *in vitro*, a recent publication demonstrated that reactive oxygen species (ROS) by Nox2 regulates Notch signaling [15^▪^]. The finding might constitute an important link between blood flow and maintenance of Notch1 expression in adult arteries [8^▪▪^]. It is well accepted that flow initiates a series of events including activation of NADPH oxidases that promote generation of ROS [17]. Thus, Nox2-mediated ROS generation in iPS cells enhances Notch signaling and their resulting angiogenic potential. Importantly, it is yet to be determined whether Nox2 regulates Notch1 *in vivo* and its relative influence in the overall levels of Notch1 receptor. 

**Box 1:**
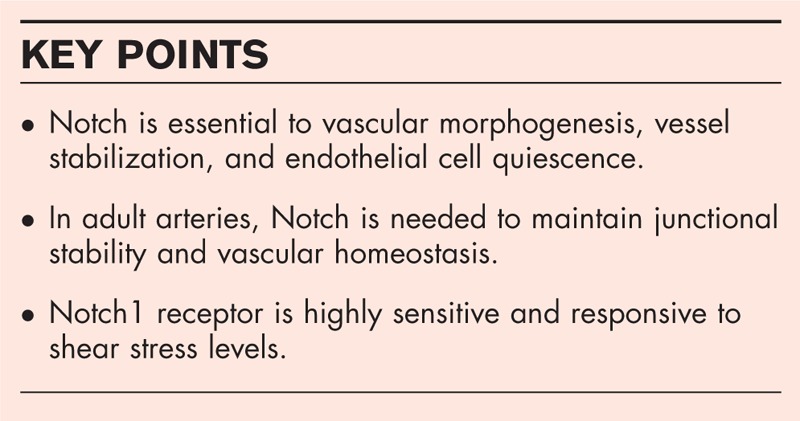
no caption available

### Arterial specification

The association of Notch expression with arteries was the first finding that linked this signaling pathway to blood vessels [18]. Importantly, inactivation of Notch1 in zebrafish impaired arterial differentiation and resulted in the ectopic expression of venous markers in the dorsal aorta [19]. This land-mark study established the essential need for Notch1 in arterial specification. More recently, transgenic lines to visualize Notch activation demonstrated the constant need for Notch signaling for the maintenance of arterial fate. In fact, reduction in Notch activity in a cell autonomous manner resulted in loss of arterial identity and incorporation of these cells into veins [20]. Although this observation was made in the context of vascular development in zebrafish, recent publications have reemphasized the requirement of continuous Notch signaling for arterial specification in mammals [9^▪▪^,^13^^▪▪^,^21^].

### Emergence and differentiation of hematopoietic stem cells from hemogenic endothelium

Previous work had shown that Notch1 was critical for hematopoietic stem cell (HSC) differentiation [22], but molecular details were lacking. As the nature of HSC budding from hemogenic endothelium gained credence, the prediction that Notch was a key participant in sorting endothelial and hematopoietic lineages was plausible yet understanding of the process only became clear recently. The essential requirement for Notch1 signaling in HSC emergence was supported by findings that global deletion of Notch1, Jag1, or RBPJ all result in embryonic lethality with associated suppression of hematopoiesis [23,24]. Importantly, these findings were partially at odds with subsequent results demonstrating that Notch was dispensable for maintenance of adult HSC and homing to the bone marrow [25]. In fact, Notch1 is not expressed in HSCs [26]. Recent work has demonstrated that although emergence of HSC requires endothelial Notch1, expression of this receptor becomes progressively reduced in HSCs as these cells depart from the hemogenic endothelium and colonize the liver [27,28]. This information has identified two types of HSCs: pre-HSC-type I with higher Notch1 levels and pre-HSC-type II with lower Notch1 levels. Definitive HSCs appear to have low or undetectable Notch at their cell surface. The prediction is that this gradual independence from Notch signals allows HSCs to gain progressive distinction from their arterial progenitors. The missing experiment is to test this prediction by artificially retaining Notch signaling in budding HSCs and assessing how this alteration affects their fate and function.

Mounting data has supported the importance of the vascular niche for homeostatic and regenerative hematopoiesis in the adult [29]. *In vitro*, the presence of ligands Jag1 and Dll4 on endothelial cells cocultured with pluripotent stems cells promotes the emergence of hematopoietic progenitors that express Notch targets Runt-related transcription factor 1 and Gata binding protein 2 [30]. This points to a critical role of Notch in driving hematopoiesis. Interestingly, endothelial Notch signaling was shown to reactivate HSC niches in aged mice, revealing not only age-dependent changes associated with Notch but also that Notch signaling reactivation in the vascular niche can enhance HSC regeneration [31^▪^].

### Notch, endothelial proliferation and junctional integrity in adult vessels

The Notch pathway was previously shown to promote endothelial cell cycle arrest in cultured monolayers [32,33]. This finding was recently expanded to reveal that shear stress upregulates Notch and facilitates flow-dependent endothelial cell quiescence through the cell cycle inhibitor cyclin-dependent kinase inhibitor 1b (Cdkn1b) and gap junction protein alpha 4 (Gja4) [9^▪▪^]. A role for Notch in endothelial cell cycle regulation and maintenance of quiescence was also supported by inducible deletion in adult mice. Here, Notch1 inactivation triggered cell cycle reentry *in vivo*[8^▪▪^] indicating that Notch1 in adult vessels provides pressure to maintain quiescence. Combined, these findings suggest that, in the context of shear stress, Notch1 is absolutely essential to maintain endothelial quiescence (Fig. 1c); however, it does not do so alone. An intricate regulatory circuitry between Bmp9 and Notch1 appears also important. Specifically, Bmp9 induces hairy and enhancer of split-related protein 1 (Hey1) and protein 2 (Hey2) and this process is disrupted by inhibition of Notch signaling [34]. Importantly, over expression of Jag1 *in vitro* resulted in an increase of endothelial cell proliferation [35]. Although the results appear to contradict the findings stated above, it is possible that modulation of one ligand might unbalance the net sum of ‘sending signals’ and thus, additional information will be required to interpret this outcome in context of findings that directly target the Notch receptor.

Notch signaling was shown to affect junctional vascular endothelial-cadherin (VE-cadherin) dynamics as evidenced by differential patterning of VE-cadherin in retinal vessels in the presence and absence of Notch activation [36]. The authors described more active/irregular patterning of adherens junctions in DAPT (*N*-[*N*-(3,5-difluorophenacetyl)-l-alanyl]-S-phenylglycine t-butyl ester, a γ-secretase inhibitor)-treated embryoid bodies and retinas, suggesting that Notch regulates cell–cell junctional rearrangements. Consistent with this finding, recent work from two independent groups has identified a role for Notch1 in promoting endothelial junctional integrity in stabilized blood vessels [8^▪▪^] and discovered that the Notch1 transmembrane domain regulates adherens junctions in microvessels under flow [37^▪▪^]. Furthermore, in the brain, Notch signaling limits vascular permeability in both resting and inflamed states [38]. The authors of this study showed that expression of both Notch and the glycosyl transferase Lfng were reduced upon inflammation suggesting that Lfng-mediated Notch glycosylation affects cell–cell contacts through altered Notch ligand binding [38]. The emerging concept is that flow forces maintain endothelial barrier through Notch.

### Notch signaling in maintenance of phenotypic identity

Hemodynamic forces are recognized regulators of gene expression in endothelial cells. Laminar flow is known to upregulate genes that are atheroprotective, whereas disturbed flow increases genes that are atheroprone [39]. In addition, flow itself can modulate arterial versus venous programs. Along these lines, exposure of endothelial-derived iPS cells to shear stress is sufficient to induce arterial specification [40]. Conversely, the application of arterial shear stress to human saphenous veins *ex vivo* leads to loss of venous identity and acquisition of arterial markers. These findings give further support to the relevance of biomechanical forces in the regulation of endothelial cell fate [41]. Interestingly, Notch1 has emerged as a mechanosensor responsible for both promoting and maintaining arterial homeostasis [8^▪▪^,^9^^▪▪^]. Notch receptor was initially recognized as an arterial marker overlapping with EphrinB2 [18], a gene later found to be regulated by Notch1. Importantly, arterial expression of Notch1 is further maintained by high shear stress leading to suppression of cell cycle and retention of arterial identity, as per expression of EphrinB2. Absence of Notch promotes arteriovenous shunts and tortuous vascular networks [42]. Collectively, the data speaks for the relevance of Notch1 levels in postnatal endothelium to maintain proper arterial, venous, and capillary organization.

In lymphatics the effects of Notch are slightly different. Notch1 limits lymphatic endothelial cell differentiation from veins and maintains lymphatic specification [43]. During lymphatic vessel sprouting, Notch1–Dll4 signaling is required for postnatal lymphangiogenesis yet Notch inhibition decreases lymphatic density, an effect attributed to differences in VEGFR3 signaling [44]. Further, in contrast to arterial endothelium, fluid flow forces in lymphatic vessels reduce Notch activity through Prox1 and promote lymphatic endothelial sprouting [45^▪^]. Prox1 is a lymphatic-specific transcription factor shown to interplay with Kruppel-like factor 2 (Klf2), a key endothelial shear stress gene. This work indicates that reduction in Notch signaling activates both blood and lymphatic endothelial sprouting and that Notch activity is modulated by shear stress. Another example of tissue context dependency for Notch signaling occurs in postnatal bone where this pathway promotes endothelial cell proliferation and vessel growth to further regulate the osteogenic aging process [46]. In the bone microenvironment, low blood flow results in reduced endothelial Notch activity with low angiogenesis and osteogenesis. Importantly, reactivation of endothelial Notch in aged mice leads to an increase in vessel-associated osteoprogenitors and more mineralized bone [47^▪^].

### Notch and inflammation

The role of endothelial Notch signaling in inflammation has been difficult to ascertain because of conflicting findings. Work has shown that proinflammatory cytokines drive changes in Notch activity to elicit a reduction in hairy and enhanced of split 1 and Hey2 leading to increased endothelial apoptosis [48]. An anti-inflammatory role was demonstrated in the bone marrow niche where Notch1 activation in bone marrow-derived endothelial cells blocked the synthesis of miR155, a microRNA involved in endothelial nitric oxide synthase downregulation and nuclear factor kappa beta (NF-κB) activation [49]. The application of high-fat diet *in vivo* or oxidized phospholipids *in vitro* was found to suppress arterial endothelial Notch1 levels, suggesting an atheroprotective role [50]. This work was later expanded to show that endothelial Notch1 was enhanced under atheroprotective flow profiles and removal of endothelial Notch1 resulted in increased atherosclerosis in a mouse model of hypercholesterolemia [8^▪▪^]. Similarly, in the endocardium, Notch signaling restricts inflammation in the regenerating heart and a loss of Notch leads to increased endothelial inflammatory gene expression and more infiltrating macrophages [51]. In contrast, Liu *et al.*[52] observed Notch pathway components to be upregulated in atherosclerotic sites and Nus *et al.*[53^▪^] showed that endothelial Jag1–RBPJ signaling promoted vascular inflammation through NF-κB and vascular cell adhesion molecule 1. Seemingly conflicting, the differences in these findings may be reflecting: the means by which the Notch pathway was inhibited (deletion of Notch1 versus RBPJ), the presence of multiple Notch receptors and ligands, and the cells probed. In fact, it can be predicted that the deletion of RBPJ would result in a different outcome as Notch signaling is mediated by both canonical (RBPJ dependent) and noncanonical (RBPJ independent) pathways.

### Notch in pathological settings

Notch activity has been linked to arteriovenous malformations where forced expression of constitutively active Notch signaling resulted in non-arterial endothelial cells developing arterial characteristics and triggering arteriovenous shunts [54,55]. Cerebral cavernous malformations have been associated with perturbed Notch signaling by cerebral cavernous malformations 1 protein-mediated induction of DLL4/Notch in endothelial cells [56]. Importantly, matrix gla protein (Mgp) maintains a balance between BMP and Notch signaling in the brain and it was shown to prevent cerebrovascular malformations [57]. The interplay between Notch and Mgp was also identified in the valve endothelium [58] where perturbations in Notch signaling led to aortic valve calcification [59,60].

In cancer, endothelial Notch1 activity was linked to metastasis by promoting a senescent, proinflammatory endothelium [61^▪^]. Yet, Notch3 activity, also in the endothelium, has been associated with limiting tumor growth in mice through induction of apoptosis [62]. As for Notch ligands in the context of cancer, Jagged, not Delta, destabilizes the tip/stalk cell phenotypes to enable poorly perfused and chaotic angiogenesis [63]. The proangiogenic role of Jagged activation of Notch signaling was also supported by findings where ligand blockade disrupted angiogenesis and inhibited tumor growth [64]. In a diabetic mouse model, Jag1 was found to be overexpressed in endothelial cells to thereby suppress Notch signaling and inhibit vascular remodeling [65].

## CONCLUSION

Few signaling molecules exert the broad and influential effect on blood vessel formation as does the Notch pathway. From the onset of vascular development, Notch determines the identity of tip and stalk cell, promotes arterial specification, and ensures the recruitment of mural cells. However, expression of Notch is not exclusive to developmental stages of the cardiovascular system, indeed, receptors and ligands continue to be present in mature vessels to maintain vascular integrity and homeostasis. Future work will likely highlight the relevance of multiple accessory molecules that act as modulators of the Notch-signaling pathway affecting protein glycosylation, ligand-receptor recognition, proteolytic cleavage, and target gene selection.

## Acknowledgements

*We would like to acknowledge the contribution of Patrick Lane in the final rendering ofFig. 1 and would like to apologize to all authors whose work could not be cited because of space limitations.*

### Financial support and sponsorship

We gratefully acknowledge funding by National Institutes of Health 2P01HL030568-31 (to MLIA).

### Conflicts of interest

There are no conflicts of interest.

## REFERENCES AND RECOMMENDED READING

Papers of particular interest, published within the annual period of review, have been highlighted as:

▪ of special interest
▪▪ of outstanding interest
